# Manipulating proteostasis to repair the F508del-CFTR defect in cystic fibrosis

**DOI:** 10.1186/s40348-016-0040-z

**Published:** 2016-03-14

**Authors:** Speranza Esposito, Antonella Tosco, Valeria R. Villella, Valeria Raia, Guido Kroemer, Luigi Maiuri

**Affiliations:** European Institute for Research in Cystic Fibrosis, Division of Genetics and Cell Biology, San Raffaele Scientific Institute, Milan, 20132 Italy; Regional Cystic Fibrosis Center, Pediatric Unit, Department of Translational Medical Sciences, Federico II University, Naples, 80131 Italy; Equipe 11 labellisée par la Ligue contre le Cancer, Centre de Recherche des Cordeliers, Paris, France; Cell Biology and Metabolomics Platforms, GustaveRoussy Comprehensive Cancer Center, Villejuif, France; INSERM, U1138, Paris, France; Université Paris Descartes, Sorbonne Paris Cité, Paris, France; Université Pierre et Marie Curie, Paris, France; Pôle de Biologie, HôpitalEuropéen Georges Pompidou, AP-HP, Paris, France; Karolinska Institute, Department of Women’s and Children’s Health, Karolinska University Hospital, Stockholm, Sweden; SCDU of Pediatrics, Department of Health Sciences, University of Piemonte Orientale, Novara, 28100 Italy

**Keywords:** Autophagy, Proteostasis, Cystic fibrosis, CFTR, CFTR-repairing therapy

## Abstract

Cystic fibrosis (CF) is a lethal monogenic disease caused by mutations in the cystic fibrosis transmembrane conductance regulator (CFTR) gene that entails the (diagnostic) increase in sweat electrolyte concentrations, progressive lung disease with chronic inflammation and recurrent bacterial infections, pancreatic insufficiency, and male infertility. Therapies aimed at restoring the CFTR defect have emerged. Thus, a small molecule which facilitates chloride channel opening, the potentiator Ivacaftor, has been approved for the treatment of CF patients bearing a particular class of rare CFTR mutations. However, small molecules that directly target the most common misfolded CFTR mutant, F508del, and improve its intracellular trafficking in vitro, have been less effective than expected when tested in CF patients, even in combination with Ivacaftor. Thus, new strategies are required to circumvent the F508del-CFTR defect. Airway and intestinal epithelial cells from CF patients bearing the F508del-CFTR mutation exhibit an impressive derangement of cellular proteostasis, with oxidative stress, overactivation of the tissue transglutaminase (TG2), and disabled autophagy. Proteostasis regulators such as cysteamine can rescue and stabilize a functional F508del-CFTR protein through suppressing TG2 activation and restoring autophagy in vivo in F508del-CFTR homozygous mice, in vitro in CF patient-derived cell lines, ex vivo in freshly collected primary patient’s nasal cells, as well as in a pilot clinical trial involving homozygous F508del-CFTR patients. Here, we discuss how the therapeutic normalization of defective proteostasis can be harnessed for the treatment of CF patients with the F508del-CFTR mutation.

Cystic fibrosis (CF), the most common lethal monogenic disease in Caucasians, is caused by mutations in the gene coding for cystic fibrosis transmembrane conductance regulator (CFTR), a 1480-amino acid protein functioning as a chloride channel at the apical membrane from the epithelial cells [1]. CF is a systemic disease, although the extent of clinical manifestations is highly heterogeneous in distinct organs [1–3]. The phenotypic consequences of the defective CFTR function comprise insufficiency of the exocrine pancreas, increased electrolytes in sweat, male infertility due to congenital absence of the vas deferens, and—most prevalent—a debilitating progressive lung disease resulting from decreased mucociliary clearance with accumulation of thick, sticky mucus, chronic inflammation, and persistent and untreatable bacterial colonization causing frequent chest infections, mainly by *Pseudomonas aeruginosa* [1–3].

Exocrine pancreatic insufficiency frequently occurs in patients with CF. It is mainly associated with “severe” CFTR mutations, where both alleles are affected by complete or major loss of function. Other gastrointestinal complications comprise recurrent abdominal pain or acute recurrent pancreatitis. Moreover, thickened secretions blocked in the bile ducts may cause progressive liver damage. Salty-tasting skin, poor growth, and poor weight gain despite normal food intake often appear in infancy, as bowel obstruction due to meconium ileus may occur in neonates. The causes of growth failure are multifactorial and include chronic lung infection, poor digestibility and absorption of nutrients through the gastrointestinal tract, and increased metabolic demand due to chronic illness.

Diagnostic procedures in CF include routine newborn screening (when blood concentrations of trypsinogen are usually measured as a surrogate marker), sweat testing, and genetic analysis. Infants with an abnormal finding in newborn screen need the sweat test to confirm the CF diagnosis. In countries in which newborn screening is not available, most individuals are primarily diagnosed by means of the sweat test, in which pilocarpine is applied to the skin to stimulate local sweating followed by iontophoresis to determine the concentration of chloride. CF can also be diagnosed by the identification of mutations in the CFTR gene.

Despite increased survival to date, several treatment methods of CF are purely symptomatic and hence fail to address the primary cause of CF, namely the loss of function of CFTR. New anti-inflammatory and antibiotic drugs are on the agenda of drug discovery approaches and clinical trials in CF patients [4].

Approximately 2000 mutations, most of which are disease relevant, have been identified in the CFTR gene and then categorized in six different classes according to their functional impact [5]. They include severe CFTR mutations that result in negligible protein synthesis (class I), misfolded mutants with defective intracellular trafficking (class II), or mutated proteins that are orthotopically expressed but exhibit impaired channel function (class III). Mutation-specific approaches aimed at correcting the CFTR defect (CFTR-repairing therapies) have recently emerged [5, 6]. These strategies are commonly focused on the identification of molecules that directly target mutant CFTR. These compounds are either capable of correcting the trafficking of CFTR mutants (“correctors”: agents that ensure the expression of the mutated protein at the apical plasma membrane) or improving channel function (“potentiators”: agents that reinstate the channel function of mutated CFTR proteins that are orthotopically expressed).

An orally available compound identified by high-throughput screening, the CFTR potentiator VX-770 (Ivacaftor, trade name Kalydeco), has been shown to efficiently reduce chloride levels in sweat and to improve lung function in CF patients harboring the G551D CFTR genotype, a rare class III CFTR mutant that affects only 4–5 % of CF patients [7, 8]. However, no effective treatments are available for the most common class II CFTR mutations. Indeed, one single mutation, p.phe508delCFTR, commonly known as F508del-CFTR (categorized in class II), accounts for about 70 % of CFTR chromosomes worldwide and is present in approximately 90 % of CF patients [5, 6]. F508del-CFTR is a misfolded protein mostly retained at the endoplasmic reticulum where it is immediately sent for degradation [9]. Despite the gating defect linked to the impaired protein conformation, F508del-CFTR shows some degree of function if rescued and stabilized at the plasma membrane (PM) [3–5]. Restoring about 20–30 % of CFTR function is believed to confer an at least partial clinical benefit to CF patients. However, although F508del-CFTR can be rescued at the PM by CFTR correctors in vitro [9], this mutant CFTR protein is unstable at the cell surface and rapidly redirected from endosomal recycling towards lysosomal delivery and degradation. The investigational F508del corrector VX-809 (Lumacaftor), which is endowed with rescuing efficacy in vitro and in primary cultures of lung cells from F508del-CFTR homozygous CF patients [9], showed only the modest efficacy in a phase II clinical trial in CF patients homozygous for the F508del-CFTR mutant [10]. Moreover, only marginal effects on lung function have been observed in phase II, and phase III randomized clinical trials aimed at testing the efficacy of a combination of the corrector Lumacaftor and the potentiator Ivacaftor [11–14]. Notably, no effects of treatment on sweat chloride, a surrogate marker of CFTR function in vivo, have been reported [12]. New combinations of correctors and potentiators are being evaluated in clinical trials in CF patients bearing F508del-CFTR mutation. However, the preclinical evidence supporting the putative effectiveness of chronic administration of the potentiator VX-770 together with a CFTR corrector (either VX-809 or VX-661) is lacking, and the mechanisms of action of these investigational correctors are still poorly understood. This suggests that interventions other than simple correction or potentiation of mutated CFTR are required in CF therapy.

## Proteostasis network is deranged in CF epithelia

Recently, more general therapeutic strategies aiming at the improvement of proteostasis have emerged [15–18]. These strategies have been designed to improve the cellular environment perturbed by the lack of a functional CFTR instead of directly targeting the mutant CFTR protein. Such a novel approach is based on recent evidence indicating that CFTR does not act as a pure ion channel but is a platform for cellular signaling within a proper cellular environment. Given the heterogeneity of signaling pathways, this new vision of CFTR implies that its function is conditioned by its cellular environment. Importantly, the protein interactomes of wild-type (WT) CFTR and the most frequent CFTR mutant, F508del-CFTR, are rather different [17, 18]. There is growing consensus that indirect measures to correct the deficient proteostasis of CFTR may be fruitful. Proteostasis modulators may reestablish the plasma membrane localization and function of F508del-CFTR by remodeling the F508del-CFTR interactome, for instance by avoiding unwanted interactions and reinstating desirable protein-protein interactions for F508del-CFTR [17]. Notably, CFTR is a key player of proteostasis in the epithelial cells, as the inhibition of CFTR function derails the intracellular environment and ignites the disposal of CFTR itself in a feed-forward loop [19].

A complex derangement of proteostasis takes place in human bronchial F508del-CFTR homozygous epithelial cell lines as well as in the lungs from F508del-CFTR homozygous (*Cftr*^*F508del*^) mice [20–23]. Dysfunctional F508del-CFTR protein induces a complex alteration of the post-translational network with increased generation of reactive oxygen species (ROS) that lead to SUMOylation with decreased ubiquitylation and persistent activation of pro-fibrotic tissue transglutaminase 2 (TG2) [20, 21]. TG2 is a versatile multifunctional protein that changes its function depending on external and internal signals [24–26]. In the presence of high Ca^2+^ levels, TG2 can act as a crosslinking enzyme, catalyzing several post-translational modifications of target proteins. At low Ca^2+^ concentrations, TG2 may function as a G-protein or as a protein disulfide isomerase, thus contributing to the functionality of mitochondrial respiratory chain complexes [24–26]. Increased levels of TG2 are observed in several human pathologies including neurodegenerative diseases such as Alzheimer’s, Huntington’s, and Parkinson’s diseases, as well as in chronic inflammatory conditions [24–26].

## Defective CFTR inhibits autophagy

TG2 overactivation in the CF epithelial cells leads to functional sequestration of Beclin 1 (BECN1) [22], a protein essential for autophagy, a mechanism required for cell survival, and involved in the pathogenesis of several human diseases [27–30]. Autophagy is pivotal in promoting cellular clearance of protein aggregates and removal of ROS sources, such as damaged mitochondria and results in the lysosomal degradation of cytoplasmic organelles or cytosolic components after their sequestration in two-membraned vesicles (autophagosomes) [27–30]. BECN1 is a haploinsufficient tumor suppressor protein essential for autophagosome formation. BECN1 dissociates from Bcl-2 during stress conditions, such as starvation, thus promoting autophagy. Subsequently, BECN1 interacts with the class III phosphatidyl-inositol 3 kinase (PI3K), human vacuolar protein sorting (hVps)34, facilitating its activation. The ER-associated class III PI3K activity is crucial for the initiation of autophagosome formation [27–30].

We discovered that human and mouse CF airways exhibit a defect in autophagy, as indicated by reduced autophagosome formation, and the accumulation of sequestrosome 1 (SQSTM1) [22], a major autophagic substrate also known as p62 [31]. This occurs in spite of the normal expression of major autophagy genes [22]. A defective autophagic response to bacterial infection has also been reported in murine CF macrophages in which reduced autophagosome formation promotes the survival of *Burkholderia cenocepacia*, as well as the pro-inflammatory hypersecretion of IL-1β [32, 33].

## Restoration of autophagy circumvents F508del-CFTR defect

Genetic depletion of SQSTM1, transgene-enforced BECN1 overexpression, or addition of autophagy-stimulatory proteostasis regulators, such as cystamine (or its reduced form cysteamine), can increase the expression level of F508del-CFTR protein and restore its function at the PM, either in CFTR homozygous CFBE41o- bronchial epithelial cell lines or in primary nasal epithelial cells freshly collected from CF patients bearing F508del-CFTR mutation, as well as in the lungs from *Cftr*^*F508del*^ mice [34, 35]. As a consequence, restoration of autophagy reduces lung inflammation in *Cftr*^*F508del*^ mice. Notably, cysteamine has a long-lived action because this drug is able to allow F508del-CFTR to reside at the PM after rescue for 24 h beyond its washout [34, 35]. These long-term effects of cysteamine are abrogated if CFTR function is simultaneously inhibited, or CFTR is depleted during washout [34, 35]. Importantly, the effects of cysteamine in reducing lung inflammation extend up to several days in vivo in *Cftr*^*F508del*^ mice, unless autophagy is inhibited by 3-methyl-adenine (3-MA) after cysteamine withdrawal [34, 35]. This suggests that the restoration of a proper autophagy flux can interrupt the CFTR-driven vicious cycle that derails the proteostasis network as, once stabilized at the epithelial surface, F508del-CFTR can sustain its own PM residence and function.

## Proteostasis regulators prevent intestinal manifestations in CF mice

In pigs and mice, the loss of the function mutations of *Cftr* causes a predominantly intestinal phenotype. Thus, 50 to 90 % of *cftr* KO and up to 40 % of *Cftr*^*F508del*^ mice die within the first 4 weeks after birth, mostly due to intestinal obstruction, unless they are kept under a special diet that reduces the risk of obstruction [35]. We observed that oral treatment for 5 weeks with cysteamine significantly reduced mortality, improved weight gain, and increased the expression of functional CFTR protein at the intestinal level, at the same time, that it restored BECN1 protein expression to wild-type levels. Such a treatment is also capable of reducing lung inflammation [35].

## Proteostasis regulators increase F508del-CFTR stability

Ensuring stability to the rescued F508del-CFTR mutant is a major concern in CFTR repairing therapies, as this mutant is rapidly dismissed from the PM after rescue and directed towards lysosomal degradation. Recent strategies aim at overcoming this issue through a combination of corrector and potentiator molecules, the latter aimed at enhancing channel activity after rescue. However, Cholon et al. and Veit et al., showed that the chronic administration of potentiators, such as Ivacaftor, is detrimental to F508del-CFTR stability, as it instead favors PM disposal of the mutant protein [36, 37]. Indeed, the mechanisms responsible for the premature F508del-CFTR removal from the PM after rescue are not fully understood. The rescued F508del-CFTR undergoes CHIP-mediated PM ubiquitination [38] and then is targeted by the ubiquitin-binding protein SQSTM1/p62, which accumulates at the PM as the consequence of disabled autophagy [34, 35]. The resulting complex composed by F508del-CFTR and SQSTM1/p62 is directed to the lysosomes for degradation [34, 35]. Moreover, defective PI3K complex III activity, due to functional sequestration of BECN1, impairs CFTR recycling, thus enhancing CFTR disposal.

Besides these autophagy-dependent mechanisms, other perturbations of the proteostasis network can influence F508del-CFTR stability at the PM. Peptide fragments released from proteolytically cleaved F508del-CFTR provoke an overactivation of a pleiotropic protein kinase (CK2), which in turn contributes to the fragmentation and poor stability of F508del-CFTR [39, 40]. The inhibition of overactive CK2 by specific inhibitors, such as CX-4945, or by means of natural compounds with known safety profile, as the over-the-counter flavonoid epigallocatechin gallate (EGCG), contributes to confer PM stability to F508del-CFTR mutant, thus prolonging the persistence of the mutant F508del-CFTR protein at the epithelial surface after rescue by cysteamine [35].

## Proteostasis regulators can restore F508del-CFTR function in CF patients

Prompted by these pre-clinical results, we recently launched clinical pilot trials to evaluate the combination of cysteamine, already approved for patients with cystinosis (FDA number NDA020392) [41], and EGCG for the treatment of F508del-CFTR homozygous patients [30]. In this phase 2 trial, cysteamine plus EGCG restored autophagy and improved CFTR function while rescuing the expression of mature (band C) CFTR protein from the nasal epithelial cells in vivo. Notably, the functional rescue of F508del-CFTR is coupled to, and correlated with, a decrease of chloride concentrations in sweat, as well as a reduction of the abundance of inflammatory cytokines in the sputum [35]. These findings indicate that such a combination treatment acts “on target”. They also indicate that it is feasible to correct the F508del-CFTR defect by manipulating the proteostasis network, thus rescuing the mutant CFTR protein and then normalizing its PM stability.

## Conclusions

We believe that any CF treatment should aim at rescuing the expression and function of functional CFTR and hence target the true causes of the disease rather than its signs. Manipulation of proteostasis network offers new perspectives for the design of candidate drugs aimed at repairing the primary CFTR defect, well beyond the idea that such molecules must directly interact with mutant CFTR protein. The proteostasis network is unique to each cell type and tissues. Thus, finding appropriate proteostasis regulators implies a profound understanding of the pathogenic mechanisms operating within each peculiar cellular context. Based on our experience, we recommend a translational research approach that comprises studies on appropriate animal models and freshly collected primary cells from CF patients to develop a strong pre-clinical rationale before evaluating drugs in the clinic. Finally, we speculate that proteostasis modulators might represent an attractive approach to other human conformational diseases, including some neurodegenerative disorders, beyond CF.
